# Dancing with death at pH 6.8 and lactate of 29 mmol/L: extreme survival in severe metformin-associated lactic acidosis - a case report

**DOI:** 10.3389/fmed.2025.1604360

**Published:** 2025-10-16

**Authors:** Said Kortli, Roland Amathieu, Mohamed Ghalayini

**Affiliations:** ^1^Intensive Care Unit, Gonesse General Hospital, Paris, France; ^2^UFR SMBH (Santé, Médecine, Biologie Humaine), Université Sorbonne Paris Nord, Bobigny, France

**Keywords:** lactic acidosis, acute kidney injury, metformin-intoxication, hemodialysis (HD), extreme survival

## Abstract

Lactic acidosis is a serious metabolic disorder characterized by an accumulation of lactate in the body, which can lead to a severe acid-base imbalance. Metformin-associated lactic acidosis is a rare but life-threatening complication of metformin therapy, particularly in the setting of acute kidney injury or other conditions that impair lactate clearance. In this case report, we present the remarkable survival of a patient who experienced severe metformin-associated lactic acidosis with a blood pH of 6.8 and a lactate level of 29 mmol/L, which are typically considered incompatible with life.

## 1 Introduction

Metformin is a first-line pharmacological treatment for type 2 diabetes mellitus (T2DM) due to its efficacy in improving glycemic control, weight neutrality, and low risk of hypoglycemia (1). It works primarily by inhibiting hepatic gluconeogenesis and improving insulin sensitivity, with most patients tolerating the drug well. However, in rare cases, metformin can lead to a life-threatening condition known as metformin-associated lactic acidosis (MALA). MALA is characterized by severe lactic acidosis, often defined by hyperlactatemia (lactate > 5 mmol/L), profound acidemia (pH < 7.35), and an elevated anion gap (2). Though rare, MALA carries a high mortality rate, with estimates ranging from 30 to 50% in reported cases (3).

The pathophysiology of MALA is multifaceted and involves the accumulation of metformin in the setting of impaired renal clearance, leading to mitochondrial dysfunction and increased lactate production (4). Unlike other forms of lactic acidosis, the primary driver in MALA is not tissue hypoxia but rather the inhibition of mitochondrial oxidative phosphorylation and gluconeogenesis, leading to reduced lactate utilization by the liver (5). This makes MALA distinct from other causes of lactic acidosis, such as sepsis or ischemia, where hypoperfusion and anaerobic metabolism dominate.

Certain risk factors predispose patients to MALA. Acute kidney injury (AKI) or chronic kidney disease (CKD) is one of the most significant risk factors, as metformin is predominantly eliminated renally through glomerular filtration and tubular secretion (6). Other contributing factors include conditions that increase lactate production or impair its clearance, such as sepsis, hypoxia, heart failure, or hepatic dysfunction (7). While lactic acidosis is a known complication of critical illness, the presence of metformin exacerbates the metabolic derangements due to its direct effects on mitochondrial function.

Although severe lactic acidosis with pH < 7.0 and lactate > 20 mmol/L is often deemed incompatible with life, there are rare reports of survival with aggressive supportive care (8).

In this report, we detail the extraordinary survival of a patient with MALA who presented with extreme acidemia (pH 6.8) and hyperlactatemia (lactate 29 mmol/L)—values rarely associated with recovery. This case emphasizes the importance of early recognition, a thorough understanding of MALA’s unique pathophysiology, and the need for timely multimodal therapeutic strategies, including renal replacement therapy and hemodynamic stabilization.

## 2 Case presentation

A 52-year-old woman with a history of poorly controlled type 2 diabetes mellitus (HbA1c 17%) and hypertension was admitted to the emergency department with complaints of progressive fatigue and generalized weakness over the preceding 48 h. Her medical history included long-term metformin therapy (2 g/day) for diabetes and antihypertensive treatment with amlodipine. She reported poor adherence to diabetes management and dietary recommendations. On further questioning, she denied recent gastrointestinal symptoms, chest pain, or significant alcohol intake but noted reduced oral intake over the previous days due to malaise.

### 2.1 Initial assessment

On admission, the patient appeared lethargic and drowsy. Vital signs revealed hypotension (blood pressure 85/55 mmHg), tachycardia (heart rate 110 beats per minute), tachypnea (respiratory rate 28 breaths per minute), and hypothermia (core body temperature 34 °C).

Capillary blood glucose testing revealed hypoglycemia (3.2 mmol/L), and clinical examination was notable for cold extremities, delayed capillary refill, and dry mucous membranes, consistent with dehydration and circulatory shock. No focal signs of infection were evident upon physical examination, although a urine dipstick test showed leukocyturia and nitrituria.

### 2.2 Laboratory investigations revealed severe metabolic derangements

**Arterial Blood Gas (ABG)**: pH 6.78, pCO_2_ 19 mmHg, bicarbonate 8.5 mmol/L, base excess −24 mmol/L.

**FIGURE 1:**
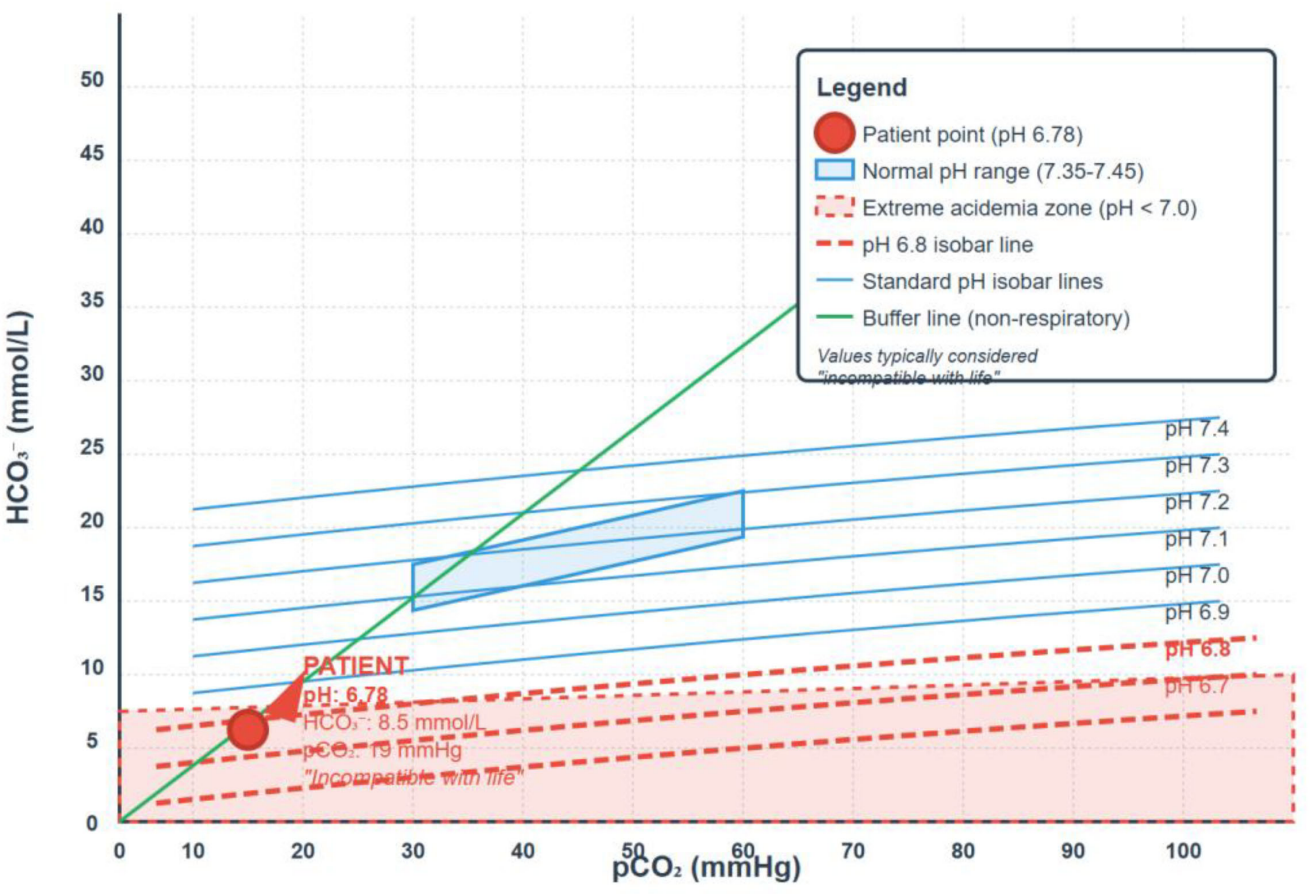
The Davenport diagram illustrating the patient’s arterial acid–base values.

The Davenport diagram (Figure 1), which illustrates the patient’s arterial acid–base values (pH 6.78, HCO_3_^–^ 8.5 mmol/L, PCO_2_ 19 mmHg), shows that her data point lies outside the expected isocurves. This deviation may reflect a potential measurement error in the reported CO_2_ and/or pH values (from which HCO_3_^–^ is calculated), rather than a limitation of the standard bicarbonate-CO_2_ buffering model. Additionally, any unmeasured anion gap would manifest as a corresponding reduction in HCO_3_^–^.

**Serum Lactate:** 29 mmol/L (reference range: 0.5–2.0 mmol/L).

**Electrolytes:** Sodium 133 mmol/L, potassium 5.9 mmol/L, chloride 102 mmol/L.

**Renal Function:** Serum creatinine 3.2 mg/dL (baseline 0.9 mg/dL), blood urea nitrogen (BUN) 58 mg/dL, consistent with acute kidney injury (AKI).

**Liver Function Tests:** Within normal limits.

**Complete Blood Count:** Leukocytosis (14,000/μL) with neutrophil predominance, hemoglobin 12.5 g/dL, platelets 210,000/μL.

**Other Tests:** Normal troponin levels, negative blood cultures initially.

**Urine Analysis:** Pyuria and bacteriuria, later confirmed as *Klebsiella pneumoniae* on culture.

The clinical presentation was characterized by severe metabolic acidosis (pH 6.78), profound hyperlactatemia (29 mmol/L), and AKI in the context of metformin use, strongly indicative of metformin-associated lactic acidosis (MALA). This diagnosis was confirmed by a markedly elevated serum metformin concentration of 100 μg/mL (normal < 10 μg/mL).

Table 1 summarizes the progression of key laboratory parameters from admission (Day 0) to post-recovery (Day 10). By Day 10, all values, including lactate, serum bicarbonate, and creatinine, had normalized or approached baseline, reflecting resolution of metabolic derangements and restoration of renal function. Liver function tests remained normal throughout the clinical course, and serial troponin measurements were consistently unremarkable. Initial blood cultures showed no growth, while urinalysis demonstrated pyuria and bacteriuria, with subsequent culture identifying *Klebsiella pneumoniae*.

**TABLE 1:** Comparison of key laboratory parameters on admission (Day 0) and after clinical recovery (Day 10), with corresponding normal reference ranges.

Parameters	On admission (Day 0)	After recovery (Day 10)	Normal range
pH	6.78	7.40	7.35 – 7.45
Lactate (mmol/L)	29	1.2	0.5 – 2.0
Bicarbonate (mmol/L)	29	24.0	22 – 28
Creatinine (mg/dL)	1.66	0.95	0.6 – 1.2
BUN (mg/dL)	21	13.2	7 – 20
K+ (mmol/L)	5.9	4.2	3.5 – 5.0
Na+ (mmol/L)	133	139	135 – 145
WBC (/μL)	14,000	7,200	4,000 – 11,000

BUN, blood urea nitrogen; WBC, white blood cell count.

The patient’s clinical course highlights the hallmark features of MALA, including severe metabolic acidosis, hyperlactatemia, and AKI, which resolved with prompt intervention. By Day 10, the patient achieved full clinical recovery, with normalization of inflammatory markers and laboratory parameters.

### 2.3 Clinical course and management

The patient was admitted to the intensive care unit (ICU) for further management. Given the severity of her presentation, a multimodal therapeutic approach was initiated:

#### 2.3.1 Hemodynamic stabilization

Aggressive fluid resuscitation with isotonic crystalloids was initiated to correct hypovolemia and improve tissue perfusion.

Aggressive fluid resuscitation was initiated with isotonic crystalloids (normal saline), with an initial bolus of 2 liters administered in the first 6 h. Norepinephrine infusion was commenced at 2 h post-admission, starting at 0.1 μg/kg/min and titrated to a maximum dose of 5.64 μg/kg/min to maintain mean arterial pressure above 65 mmHg. The high vasopressor requirements reflected the profound distributive shock characteristic of severe MALA, where acidemia-induced myocardial depression and vasodilation compromise hemodynamic stability.

Norepinephrine titration followed a protocolized approach based on hemodynamic response and lactate clearance. Peak vasopressor requirements occurred within the first 24 h, after which progressive down-titration was possible as metabolic acidosis corrected and cardiac contractility improved. By 72 h, vasopressor support was completely discontinued, reflecting restoration of cardiovascular function and resolution of distributive shock.

#### 2.3.2 Renal replacement therapy (RRT)

Early and prolonged intermittent hemodialysis was initiated within 6 h of ICU admission, with a total duration of 20 h. This therapy aimed to remove accumulated metformin, correct acidemia, and improve lactate clearance. Dialysis fluid was bicarbonate-buffered to help restore metabolic balance.

Our patient received 20 h of intermittent hemodialysis using bicarbonate-buffered dialyzate (bicarbonate concentration 35 mmol/L) to optimize acid-base correction. The choice of intermittent hemodialysis over continuous renal replacement therapy (CRRT) was based on its superior metformin clearance capacity and rapid correction of severe acidemia. Studies demonstrate that intermittent hemodialysis achieves metformin clearance rates of 200–500 mL/minute compared to 50 mL/minute with CRRT (9).

#### 2.3.3 Empirical antibiotic therapy

Empirical broad-spectrum antibiotics (piperacillin-tazobactam 4 g IV every 6 h and amikacin 30 mg/kg daily) were initiated at 12 h to address the confirmed *Klebsiella pneumoniae* urinary tract infection. This intervention was crucial as sepsis represents a common precipitating factor for MALA and contributes to ongoing lactate production through tissue hypoperfusion.

#### 2.3.4 Metabolic monitoring and RRT adjustment

Continuous monitoring of arterial blood gases every 4 h during the first 24 h guided RRT intensity and duration. The bicarbonate-buffered dialyzate concentration was maintained at 35 mmol/L throughout treatment to optimize acid-base correction without precipitating metabolic alkalosis. Lactate clearance served as the primary endpoint for RRT efficacy, with treatment continued until lactate normalized to <2 mmol/L.

#### 2.3.5 Pharmacological transitions

Metformin therapy was permanently discontinued upon diagnosis confirmation. The patient was transitioned to insulin therapy using a continuous infusion protocol (starting at 0.1 units/kg/hour) to maintain glucose control during the acute phase, with subsequent conversion to subcutaneous insulin before discharge. This transition acknowledges the absolute contraindication to metformin rechallenge following MALA.

#### 2.3.6 Additional supportive measures

Hypoglycemia was managed with continuous dextrose infusion until stabilization of blood glucose levels.

Electrolyte imbalances were closely monitored and corrected (including hyperkalemia).

#### 2.3.7 Renal function recovery

Serial monitoring of serum creatinine demonstrated progressive improvement from the peak of 3.2 mg/dL to baseline values within 10 days. The rapid renal recovery likely reflects the reversible nature of acute tubular necrosis secondary to hypoperfusion rather than metformin-induced nephrotoxicity, as metformin itself does not cause direct kidney injury.

These comprehensive interventions, implemented in a coordinated, time-sensitive manner, demonstrate the importance of multimodal therapy in MALA management. The successful outcome despite extreme metabolic derangements underscores the potential for recovery when evidence-based protocols are rigorously applied in this life-threatening condition.

#### 2.3.8 Outcome and follow-up

**FIGURE 2:**
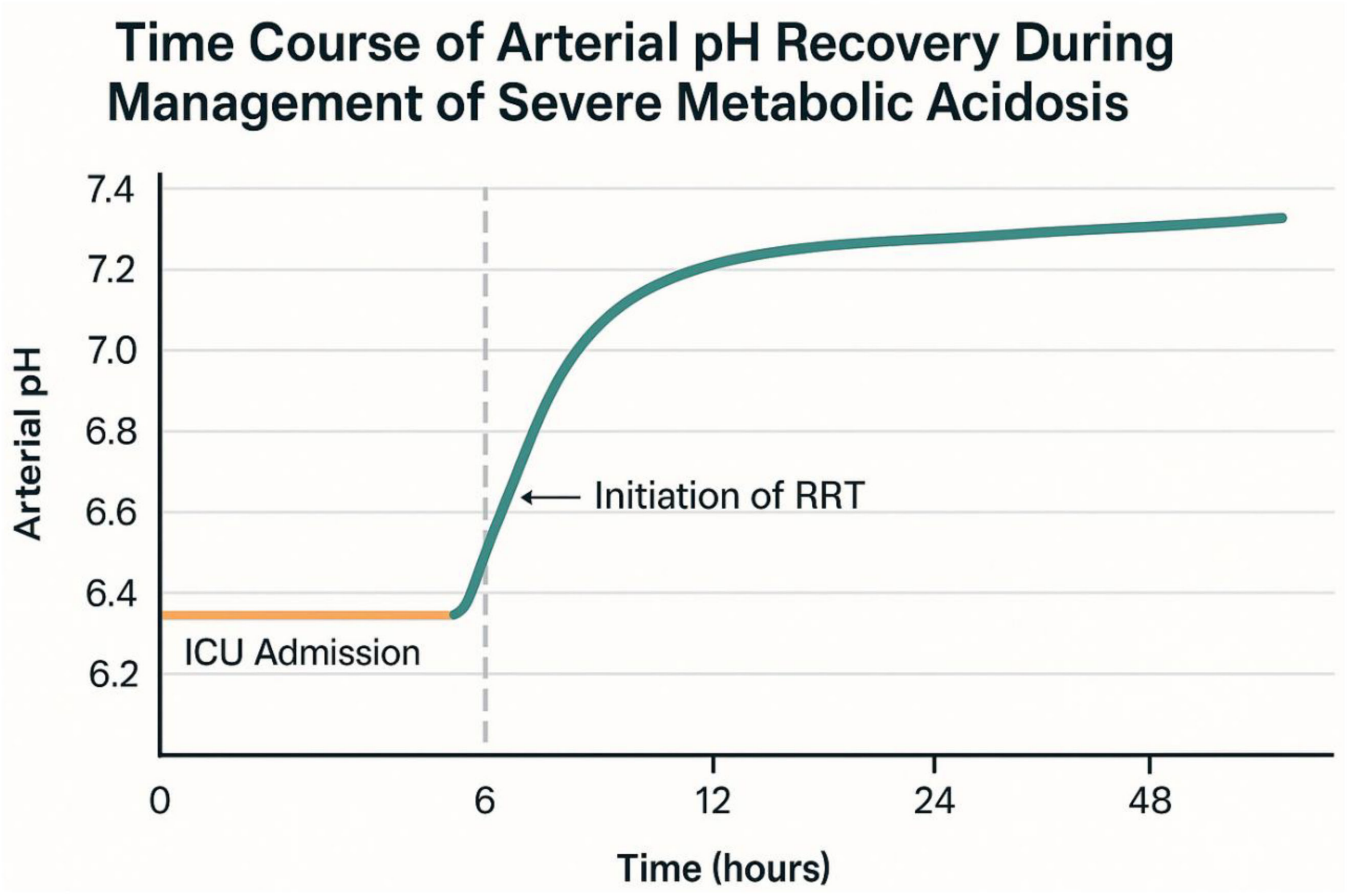
Time course of arterial pH recovery during management of severe acidemia.

**FIGURE 3:**
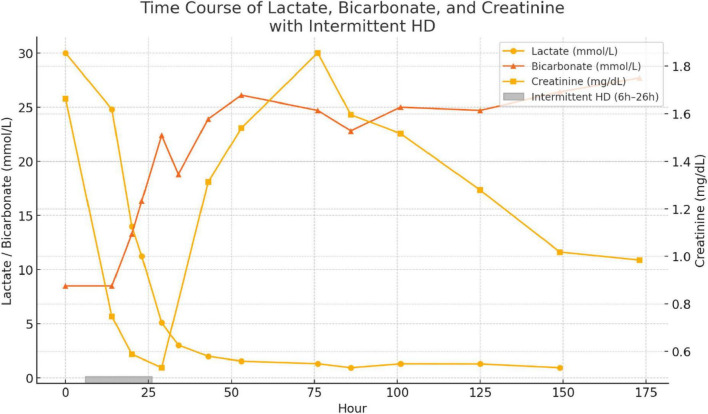
Time course of plasma lactate (circles), serum bicarbonate (triangles) and serum creatinine (squares) from admission (Hour 0) through Hour 173. The shaded bar just above the x axis denotes the 20-h intermittent hemodialysis session initiated at Hour 6.

Despite the severity of her metabolic derangements (pH 6.78 and lactate 29 mmol/L), the patient showed remarkable improvement within 48 h. Key milestones in her recovery included:

- Progressive hemodynamic stabilization with down-titration of norepinephrine.
- Arterial pH demonstrated progressive recovery from the initial value of 6.78, with marked improvement following initiation of renal replacement therapy at 6 h post-admission (Figure 2).
- By day 3, vasopressor support was discontinued, and renal function began to recover.
- Metformin was permanently discontinued due to the risk of lactic acidosis associated with acute renal impairment, and a basal-bolus insulin regimen was initiated. This regimen included glargine (0.2 IU/kg at bedtime) and aspart (0.05 IU/kg before meals), with the goal of achieving fasting blood glucose levels of 6–8 mmol/L and postprandial levels below 10 mmol/L. Prior to discharge on Day 17, the patient received comprehensive education on insulin injection techniques, self-monitoring of blood glucose (4–6 capillary measurements daily), and individualized dietary and lifestyle counseling. Figures illustrating the biological progression mirrored the clinical improvement:

The patient was successfully weaned off mechanical support and discharged from the intensive care unit (ICU) on Day 5. Intermittent hemodialysis was initiated 6 h after admission and continued for a total of 20 h, resulting in significant improvement in metabolic and renal parameters (Figure 3). Plasma lactate levels, which were critically elevated at admission (30 mmol/L), decreased rapidly to 1.0 mmol/L by Hour 86. Concurrently, serum bicarbonate levels rose from 8.5 mmol/L to 24.7 mmol/L, reflecting correction of severe metabolic acidosis. Serum creatinine peaked at 164 μmol/L (1.92 mg/dL) at Hour 76 but gradually declined to 99 μmol/L (1.12 mg/dL) by Hour 173. Acid–base balance normalized by Day 4, and hemodynamic stability was achieved, allowing for vasopressor support to be discontinued by Day 3.

**FIGURE 4:**
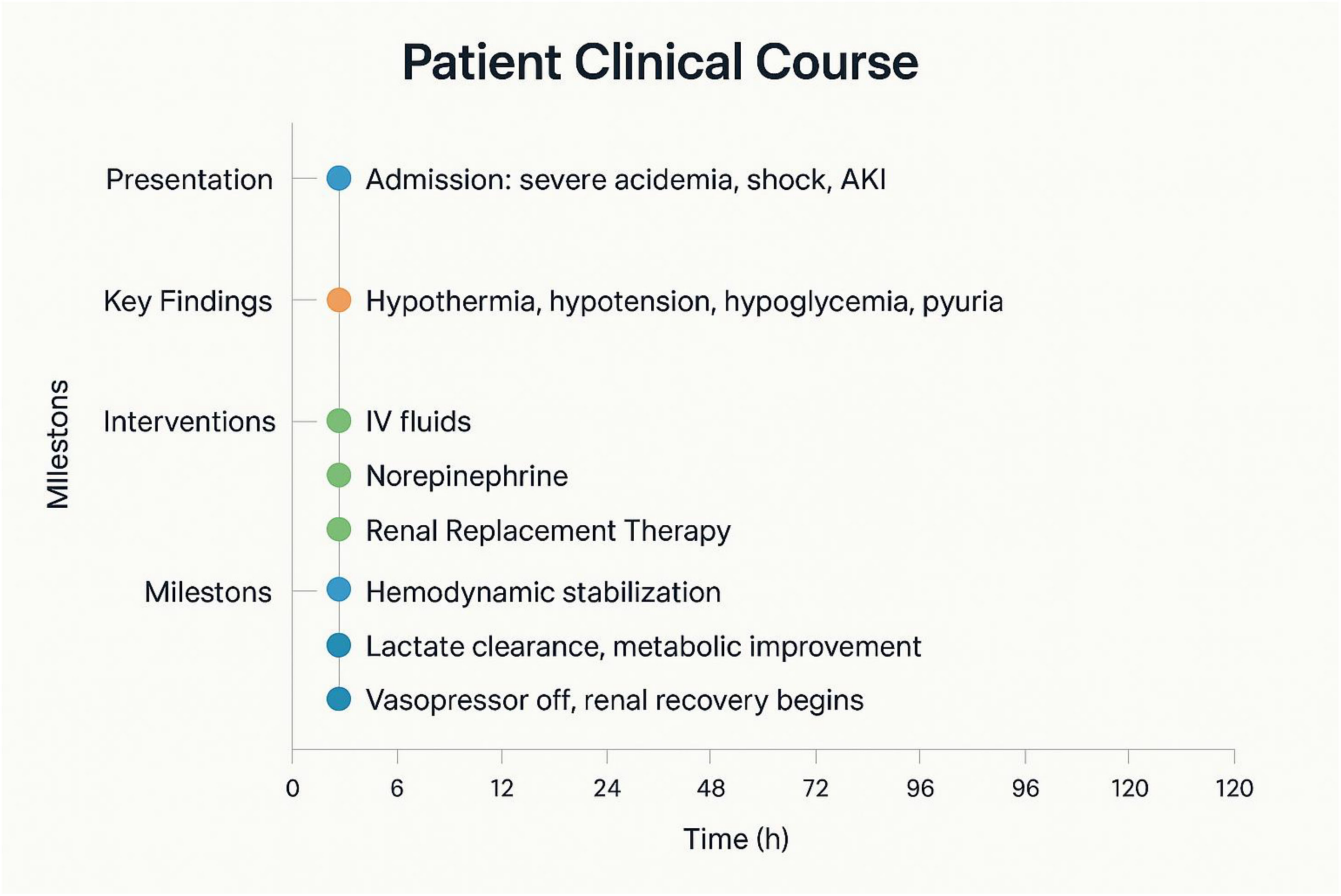
Timeline chart summarizing the patient’s clinical presentation, key findings, therapeutic interventions, and recovery milestones over 120 h.

The comprehensive clinical timeline, including presentation, key findings, therapeutic interventions, and recovery milestones over the first 120 h, is illustrated in Figure 4. By Day 10, all key laboratory values, including pH, lactate, bicarbonate, and creatinine, had returned to or near normal ranges (Table 1), permitting the cessation of renal replacement therapy. Metformin therapy was permanently discontinued, and the patient was transitioned to an insulin-based regimen. She was counseled on the importance of strict glycemic control, adherence to treatment protocols, and the necessity of regular follow-up care. The patient’s recovery trajectory highlights the effectiveness of timely hemodialysis and supportive care in resolving metabolic derangements and achieving clinical stability.

This case highlights the potential for survival in MALA, even in the presence of extreme acidemia and hyperlactatemia, when aggressive and timely interventions are implemented.

## 3 Discussion: pathophysiology of metformin-associated lactic acidosis (MALA)

Metformin-associated lactic acidosis (MALA) is a rare but severe complication of metformin therapy, characterized by the accumulation of lactate and profound acidemia. Its pathophysiology involves a complex interplay of mitochondrial dysfunction, impaired lactate clearance, and predisposing clinical conditions.

### 3.1 Mitochondrial dysfunction induced by metformin

Metformin primarily acts by inhibiting hepatic gluconeogenesis through suppression of mitochondrial respiratory chain complex I. This inhibition reduces ATP production and shifts cellular metabolism toward anaerobic glycolysis, resulting in increased production of lactate (10). Under normal circumstances, lactate is utilized by the liver for gluconeogenesis. However, metformin’s suppression of gluconeogenesis leads to reduced lactate consumption, further contributing to its accumulation.

Additionally, at high plasma concentrations, metformin directly impairs mitochondrial oxidative phosphorylation, exacerbating lactate production and reducing the cell’s ability to buffer acidemia. This mechanism is particularly pronounced in the liver and skeletal muscle, where mitochondrial energy demands are high (1).

### 3.2 The role of renal dysfunction

Metformin is predominantly cleared by the kidneys through glomerular filtration and active tubular secretion. In the setting of acute kidney injury (AKI) or chronic kidney disease, reduced clearance of metformin leads to its accumulation, amplifying its inhibitory effects on mitochondrial respiration (2).

Furthermore, renal dysfunction impairs lactate clearance, as the kidneys contribute up to 30% of lactate metabolism under normal conditions (3). This dual impairment—metformin accumulation and reduced lactate metabolism—creates a vicious cycle that accelerates the development of severe lactic acidosis.

### 3.3 Acidosis and the impact on cellular function

The profound acidemia observed in MALA (pH < 7.0) has significant physiological effects:

- **Hemodynamic instability:** Acidosis impairs myocardial contractility and systemic vascular tone, contributing to distributive and cardiogenic shock (4). This exacerbates tissue hypoperfusion and further promotes anaerobic metabolism and lactate production.
- Intracellular dysfunction: A low intracellular pH disrupts enzymatic activity and ion gradients, compounding mitochondrial dysfunction and impairing cellular recovery mechanisms.

### 3.4 Triggers and predisposing factors

While metformin alone rarely causes lactic acidosis, precipitating factors such as AKI, sepsis, or hypoxia are almost always involved. In this case, the urinary tract infection with *Klebsiella pneumoniae* likely contributed to systemic inflammation and sepsis-related hypoperfusion, further increasing lactate production. The patient’s poorly controlled type 2 diabetes (HbA1c 17%) may have also predisposed her to metabolic stress and reduced lactate clearance.

### 3.5 Reframing the lactate threshold in MALA

Although lactate levels > 20 mmol/L and pH < 6.8 are traditionally considered incompatible with life, MALA appears to have a unique pathophysiology compared to other forms of lactic acidosis, such as those seen in sepsis or ischemia. In MALA, the predominant mechanism is impaired lactate clearance and mitochondrial dysfunction, rather than overwhelming lactate production secondary to tissue hypoxia. This distinction may partly explain the potential for survival in extreme cases, provided aggressive supportive therapy is initiated promptly (5).

### 3.6 Role of renal replacement therapy

Renal replacement therapy (RRT) plays a dual role in the management of MALA:

- **Metformin elimination:** Hemodialysis effectively removes metformin due to its low molecular weight and lack of protein binding.
- **Lactate and acid-base correction:** RRT helps to normalize lactate levels and correct acidemia by buffering the extracellular environment. While lactate itself is not directly removed by hemodialysis, the improvement in metabolic homeostasis and hemodynamics indirectly reduces lactate production.

## 3.7 Strengths of the current case

### 3.7.1 Extreme survival at record-breaking parameters

This case represents one of the most severe documented survivals of MALA, with a blood pH of 6.78 and lactate of 29 mmol/L—values that approach the physiological limits of life. While Kajbaf and Lalau demonstrated survival is possible with pH as low as 6.5 and lactate up to 35 mmol/L, such extreme cases remain exceptionally rare in literature.

Most reported MALA cases involve less severe acidemia, such as the case reported by Mahmood et al. (pH 6.57, lactate 16.3 mmol/L) or the case by Rodríguez-Villar et al. (pH 7.042, lactate 20.0 mmol/L).

### 3.7.2 Multidisciplinary care excellence

The case exemplifies the critical importance of coordinated time-sensitive interventions in MALA management. The successful outcome was achieved through seamless integration of emergency medicine, intensive care, nephrology, and pharmacy expertise. Early recognition within 6 h of admission, immediate initiation of renal replacement therapy, and aggressive hemodynamic support demonstrate the potential for survival when evidence-based protocols are rigorously implemented.

### 3.7.3 Novel insights into MALA pathophysiology

This case provides valuable insights into the unique pathophysiology of MALA compared to other forms of lactic acidosis. Unlike sepsis-related acidosis where tissue hypoxia predominates, this patient’s survival despite extreme acidemia supports the concept that MALA involves primarily impaired lactate clearance rather than overwhelming lactate production. The patient’s-maintained consciousness despite pH 6.78 contrasts sharply with typical expectations, where altered mental status typically occurs at pH < 6.9.

## 3.8 Limitations and methodological considerations

### 3.8.1 Single case report limitations

As an isolated case report, this study has inherent limitations in generalizability and statistical power. The exceptional nature of survival at these extreme parameters may represent an outlier rather than a reproducible outcome, limiting the ability to extrapolate management strategies to other patients with severe MALA.

### 3.8.2 Absence of long-term follow-up data

While the patient achieved complete renal recovery within 10 days and was discharged on day 5, the manuscript lacks extended follow-up data beyond immediate hospitalization. Long-term neurological outcomes, cardiovascular sequelae, and diabetes management strategies are not addressed. Studies suggest that MALA survivors generally have good long-term outcomes, but individual variation exists.

### 3.8.3 Missing pharmacokinetic data

The case lacks detailed pharmacokinetic analysis of metformin clearance during dialysis, which would provide valuable insights for optimizing RRT protocols. While the serum metformin level was elevated at 100 μg/mL, serial measurements during dialysis would have enhanced understanding of clearance kinetics and treatment adequacy.

### 3.8.4 Limitation of the bicarbonate-CO_2_ buffering model

One limitation of this case is that the reported values for pH, CO_2_, and HCO_3_^–^ do not meet the expected equilibrium conditions for the carbonic acid-bicarbonate buffer system. Given the low magnitude of fluxes in physiological systems, the likelihood of this buffer system being out of equilibrium is minimal. Therefore, these deviations may be attributed to either measurement error or calculation discrepancies. While this does not undermine the overall clinical findings, it highlights the need for cautious interpretation of extreme values in acid-base disturbances.

### 3.8.5 Limited mechanistic investigation

The manuscript does not explore potential genetic or metabolic factors that might have contributed to this patient’s remarkable survival. Individual variation in mitochondrial function, lactate metabolism, or acid-base buffering capacity could explain exceptional outcomes but were not investigated.

## 4 Literature context and comparative analysis

### 4.1 Treatment modality comparisons

This case utilized 20 h of intermittent hemodialysis, contrasting with other successful approaches in the literature. Keller et al. reported success with continuous renal replacement therapy (CRRT) in six patients with severe MALA (mean pH 6.92, lactate 14.4 mmol/L), achieving survival through high-volume hemofiltration (6). Gatti et al. demonstrated improved mortality rates (21.4%) using sustained low-efficiency dialysis (SLED) in 28 patients, suggesting multiple effective dialytic approaches (7).

The choice between intermittent hemodialysis and CRRT remains debated. While this case achieved success with intermittent hemodialysis, other reports suggest CRRT may be superior for hemodynamically unstable patients (8). The key appears to be early initiation rather than specific modality selection.

### 4.2 Mortality rate context

The survival in this case is particularly remarkable given historical mortality rates in severe MALA. Early case series reported mortality rates of 30%–50%, while more recent series with aggressive management show improved outcomes. Gatti et al. achieved 21.4% mortality in their SLED series, with all deaths occurring within 24 h (7). This temporal pattern suggests that patients surviving the initial critical period have excellent prognosis, as demonstrated in our case.

### 4.3 Precipitating factor analysis

Unlike many MALA cases precipitated by acute dehydration (86.4% in Gatti’s series) or sepsis, this patient’s primary trigger was urinary tract infection with *Klebsiella pneumoniae* (7). The combination of poor glycemic control (HbA1c 17%) and medication non-adherence likely contributed to her susceptibility, highlighting the importance of patient education and regular monitoring.

### 4.4 Clinical presentation variability

The patient’s preserved consciousness despite extreme acidemia contrasts with typical expectations. Most literature describes progressive mental status changes with severe acidosis, yet remarkable cases exist of patients maintaining alertness at extremely low pH values (11). This highlights the unpredictable nature of MALA presentation and the importance of not using clinical appearance alone to guide treatment intensity.

### 4.4.1 Clinical implications and lessons

This case demonstrates the critical interplay between mitochondrial dysfunction, renal impairment, and acid-base imbalance in MALA. Early recognition and aggressive intervention are paramount to interrupt the pathological cascade. The patient’s recovery highlights the importance of:

- Early initiation of renal replacement therapy to address metformin accumulation and acidemia.
- Hemodynamic stabilization with vasopressors to restore tissue perfusion and mitigate anaerobic metabolism.
- Identification and treatment of precipitating factors, such as infections, to prevent further lactate accumulation.

## 5 Conclusion

Metformin-associated lactic acidosis (MALA) is a rare but life-threatening condition with a complex pathophysiology involving mitochondrial dysfunction, impaired lactate clearance, and contributing factors such as renal impairment or sepsis. This case demonstrates that survival is possible even in the presence of extreme acidemia (pH 6.8) and hyperlactatemia (lactate 29 mmol/L)—values traditionally considered incompatible with life—when aggressive and timely multimodal interventions are implemented. Early recognition of MALA, combined with targeted therapeutic strategies such as renal replacement therapy, hemodynamic stabilization, and correction of precipitating factors, is critical for improving patient outcomes. This case underscores the importance of understanding the unique mechanisms of MALA to guide effective management and highlights the resilience of physiological systems when supported by prompt, comprehensive care.

## Data Availability

The raw data supporting the conclusions of this article will be made available by the authors, without undue reservation.
